# Identifying systemic inequity in higher education and opportunities for improvement

**DOI:** 10.1371/journal.pone.0264059

**Published:** 2022-04-08

**Authors:** Kameryn Denaro, Kimberly Dennin, Michael Dennin, Brian Sato

**Affiliations:** 1 Division of Teaching Excellence and Innovation, University of California, Irvine, California, United States of America; 2 Division of Undergraduate Education, University of California, Irvine, California, United States of America; 3 Department of Physics & Astronomy, University of California, Irvine, California, United States of America; 4 Department of Molecular Biology and Biochemistry, University of California, Irvine, California, United States of America; Northwestern University, UNITED STATES

## Abstract

It is well established that there is a national problem surrounding the equitable participation in and completion of science, technology, engineering, and mathematics (STEM) higher education programs. Persons excluded because of their ethnicity or race (PEERs) experience lower course performance, major retention, sense of belonging, and degree completion. It is unclear though how pervasive these issues are across an institution, from the individual instructor, course, and discipline perspectives. Examining over six years of institutional data from a large-enrollment, research-intensive, minority-serving university, we present an analysis of racial opportunity gaps between PEERs and non-PEERs to identify the consistency of these issues. From this analysis, we find that there is considerable variability as to whether a given course section taught by a single instructor does or does not exhibit opportunity gaps, although encouragingly we did identify exemplar instructors, course-instructor pairs, courses, and departments that consistently had no significant gaps observed. We also identified significant variation across course-instructor pairs within a department, and found that certain STEM disciplines were much more likely to have courses that exhibited opportunity gaps relative to others. Across nearly all disciplines though, it is clear that these gaps are more pervasive in the lower division curriculum. This work highlights a means to identify the extent of inequity in STEM success across a university by leveraging institutional data. These findings also lay the groundwork for future studies that will enable the intentional design of STEM education reform by leveraging beneficial practices used by instructors and departments assigning equitable grades.

## Introduction

A main consideration for educators and researchers examining means to improve higher education is the role that the institution plays in recreating and reinforcing structural inequalities. Related to this is how a student’s race and ethnicity differentially affects their experiences and outcomes in the education system [1–9]. Identifying the root cause of this discrepancy is not a simple task, due to the complex nature of structural racism and the importance of intersectionality in regards to one’s identity and the effects of this on lived experience [10].

Critical race theory (CRT) lends itself naturally to breaking down how structural racism functions at the university level. The foundations of CRT were established by Dr. Derrick Bell and Dr. Alan Freeman in the mid-1970s and developed out of legal scholarship [11–14]. The basic tenets of CRT make it so that it is interdisciplinary and can be used to critique any system. The foundation of CRT is that institutional racism is ingrained in the structures of American society, as the power structure in America was founded on white privilege and the marginalization of people of color. Institutional racism can exist without individual racists, so even though people may not be overtly acting in a racist manner, people of color are still adversely affected by the structures in society. In addition, CRT rejects the ideas of liberalism and meritocracy, which are so often tied to the education system. The educational system, and the instructors and courses within that system cannot escape the influences of institutional racism [15–21]. Without being aware of this, and consciously designing a course to attempt to minimize the effects of institutional racism, the courses instructors design are more likely to work against persons excluded because of their ethnicity or race (PEERs) [22, 23]. According to CRT, these instructors are by no means racists, they are just working within a structure that disadvantages PEERs, and without conscious effort, will continue to reproduce structures that are imbued with institutional racism in their courses.

Related to this, Asai’s commentary on race [22] includes a discussion of the disproportionately negative outcomes faced by science, technology, engineering, and mathematics (STEM) PEERs leading to the need to create “institution-centered approaches that will change the culture of science and education”. The piece [22] also discusses the negative impact of the implicit belief in the concept of “mismatch” (the idea that Black, Latinx, and indigenous persons’ underperformance is inevitable because they are less academically prepared than White and Asian students). Carnavale et. al [24] use national data to show that the mismatch theory in education is empirically wrong. This work finds that all students at more selective schools have higher graduation rates, not only the most academically prepared as defined by standardized test scores. In fact, students in the lowest test quartile have a higher graduation rate at selective research universities than students from the top test quartile who attend open-access institutions. This is true not only for students who are White, but students who are Black and Latinx as well. In our work, we also reject the concept of “mismatch” and use the lens of CRT to guide our evaluation of systemic inequity within the higher education system.

Creating educational environments that foster the academic success of PEERs is essential, as successful navigation of the education system is often imperative for advancing oneself in society [25–27]. This success is not based solely on merit, and is dependent upon the interaction of many factors [10], a prominent one being race/ethnicity. The transmission of cultural models and the resulting hierarchy within colleges and universities has resulted in the prioritizing of dominant forms of knowledge that are often foreign or less obvious to students from minoritized backgrounds [10, 26–29]. This simultaneously denies the legitimacy of other forms of cultural capital derived from different races/ethnicities and serves to prevent PEERs from attaining the same levels of success as their non-PEER counterparts. The resulting disparity in success, which we refer to as the racial opportunity gap, can be identified across a wide range of metrics including course grades, GPAs, graduation rates, and retention in STEM majors [10, 25–28, 30–32].

The challenge for higher education institutions moving forward is how to address structural racism in the education system and to implement strategies to eliminate the racial opportunity gap. Evidence-based interventions that have been successful in these efforts include learning communities [6], distance education programs [2], undergraduate research and student organizations [4], light-touch values affirmation and utility-value interventions [5, 33, 34], use of alternative assessments [35], and the effectiveness of shifting to a diversity cognitive frame [3]. Additionally, there has been increasing work on classroom practices and course structures that improve student outcomes, particularly for PEERs [1, 7–9, 36, 37]. These include the incorporation of active learning pedagogies which emphasize peer to peer or student/instructor interaction, higher order problem solving, and facilitated, independent learning time. While these studies are promising, it is clear that adoption of evidence-based teaching practices has not occurred on an institutional level [1, 7–9, 36, 37].

Because of the variability in instructor practices [38], the undergraduate STEM experience is likely to differ from course to course. This makes it probable that the previously described STEM equity issues [2–6, 33–36] are manifested to varying degrees throughout a department and across the institution. This paper seeks to establish a method to characterize the pervasiveness of equity issues across an institution and to identify STEM instructors whose courses show minimal racial opportunity gaps for PEERs. Follow up work can then identify the beneficial practices employed by these faculty and determine whether practices in one course have indirect impacts on student success in their other courses. Specifically, we aim to address the following research questions:

1. How can we identify specific STEM instructors that either do or do not consistently exhibit opportunity gaps in their course sections between non-PEER and PEER populations?
2. Using this methodology, are certain STEM departments more likely to exhibit racial opportunity gaps versus others?
3. Using this methodology, do racial opportunity gaps vary within a STEM department?

## Materials and methods

### Data

Primary analyses were performed on a dataset containing 4,644 undergraduate course sections at a selective research university in the Western US. Between the Fall 2013 and Winter 2020, we identified course sections within STEM departments that had at least 10 PEERs and 10 non-PEERs in the same course section. The inter-quartile range (IQR) for the percent of PEERs per course section in this sample was 28–49% PEERs; in other words, the middle 50% of course sections had between 28% and 49% PEERs. To control for class composition, we focus on these courses that had similar PEER representation (i.e. PEER representation within the inter-quartile range). All instructors who taught these STEM courses were included in our analyses with team taught courses being counted by marking the instructor team as a unique instructor. Instructors with joint appointments were included in the department analysis if they taught courses within that department. Overall, the data spans 4,644 course sections (905 courses, 1,983 instructors, and 2,752 course-instructor pairs) across 40 STEM departments. We define STEM courses as courses taught within a STEM department. We are using the National Science Foundation of STEM which “includes psychology and the social sciences (e.g., political science, economics) as well as the physical and life sciences and engineering (e.g., physics, chemistry, biology, mathematics)” [39].

The dataset included student demographics and transcript data including course enrollments and performance. Student demographic data included the student’s PEER status (Black, Latinx, Pacific Islander, and peoples indigenous to the spaces comprising the United States and its territories). Transcript data included information on each course (i.e. course number, subject, type, and level) and student performance (i.e. grade in course). There were no incomplete grades in the transcript data at the time of our data collection. This work was a retrospective study using institutional data. Data were accessed by one member of the study team in an identifiable form with a waived requirement for informed consent according to regulations established by the study campus’ institutional review board. All data were de-identified prior to analysis. This study was approved by the University of California, Irvine’s Institutional Review Board (IRB #2018–4211). Exclusion criteria were: (1) course sections with fewer than 10 PEERs and 10 non-PEERs, (2) course sections with less than 28% of the students being PEERs, (3) course sections with more than 49% of the students being PEERs, (4) non-STEM courses, (5) students who withdrew from the course, (6) graduate courses. Our fifth exclusion criteria was set because students who wish to withdraw must withdraw from all courses in a particular term. Therefore, we assume that the withdrawal is not related to any particular course and due to individual student circumstances.

### Statistical tests

We used two different methods to explore the differences in grades given to PEERs and non-PEERs. First, we conducted a Chi-square test of homogeneity to determine if there was a difference in the fraction of A’s and B’s awarded compared to C’s, D’s, and F’s between PEERs and non-PEERs (Δ%AB). In a study of predictive analytics for STEM success, He and colleagues [40] found that for 15 years of Biology course data, students should aim for a grade of B or better to have a higher chance of graduating as a Biology major. Second, we performed a two-sample t-test for independent samples (PEERs and non-PEERs) to test if there was a difference in the average grade received by the two groups on a 4.0 scale (ΔGP).

To test if racial opportunity gaps (between PEERs and non-PEERs) vary across an undergraduate department (i.e. lower versus upper division), we used a two-sample t-test of the differences of the course-section average ΔGP and Δ%AB. For all analyses, we compared raw grades without incorporating students’ prior academic achievement to more accurately capture the student experience. Since undergraduates in a course taught by a specific instructor do not consider their demographic characteristics or prior academic achievement when considering their course outcomes relative to their classmates, this analysis reflects student perceptions of relative performance. Similarly, future employers or graduate admission committees do not make decisions on who to employ or who to admit adjusted for previous performance.

## Results

### Research question 1: How can we identify specific STEM instructors that either do or do not consistently exhibit opportunity gaps in their course sections between non-PEER and PEER populations?

One measure of overall course performance for PEERs and non-PEERs can be determined by tabulating the numbers of A and B grades earned relative to C, D, and F grades for each population. Table 1 provides an example of these results with Instructor *J* who taught the same course five times. For example, in course section three for Instructor *J*, 172 non-PEERs received either an A or B, whereas 67 PEERs received an A or B with the percentage of PEERs receiving A and B grades being significantly lower than the percentage of non-PEERs (46% of PEERs versus 63% of non-PEERs); the difference in the percent of A and B grades given to PEERs and non-PEERs (Δ%AB) was -17%. Furthermore, while PEERs made up 35% of this course section (taught by Instructor *J*), the PEERs received only 28% of the A and B grades and received 44% of the C, D, and F grades (i.e. PEERs experienced less A and B grades than expected).

**Table 1:** Summary of the counts and proportion of students obtaining A and B grades versus C, D, and F grades for Instructor *J* across 5 sections of the same course.

Section	PEERs	AB	CDF	n	%*AB*	Δ%*AB*	χ^2^	p-value
1	No	131 (125)	91 (97)	222	59		2.36	0.1294
	Yes	44 (50)	45 (39)	89	49	-10		
	Total	175	136	311				
2	No	89 (89)	68 (68)	157	57		0.01	>0.999
	Yes	37 (37)	29 (29)	66	56	-1		
	Total	126	97	223				
3	No	172 (155)	100 (117)	272	63		12.14	0.001
	Yes	67 (84)	80 (63)	147	46	-17		
	Total	239	180	419				
4	No	167 (155)	94 (106)	261	64		7.38	0.0115
	Yes	64 (76)	65 (53)	129	50	-14		
	Total	231	159	390				
5	No	94 (81)	39 (52)	133	71		12.50	<0.001
	Yes	50 (63)	54 (41)	104	48	-23		
	Total	144	93	237				
Average						-13		

The observed and expected counts of grades (A and B grades versus C, D, and F grades) versus PEERs status for Instructor *J* are displayed for each course section and the results of each of the *χ*^2^ tests. The expected counts are in parentheses. These are determined based on the proportion of PEERs and non-PEERs in a particular course section as well as the proportion of AB and CDF grades given in the respective course section. The difference in the percent of A and B grades given to PEERs and non-PEERs (Δ%*AB*) is also displayed.

Another means to identify the presence of a racial opportunity gap is by calculating the grade point average (on a 4.0 scale) difference (ΔGP) between PEERs and non-PEERs. Table 2 provides the summary statistics and *t*-test results for the same course taught by Instructor *J*. If we again use section 3 as an example, the difference in the average grade received by PEERs and non-PEERs was -0.38, a statistically significant difference (*t* = -3.93, *p*< 0.001). Similar to the previous comparison, this test provides evidence that PEERs received lower grades on average compared to non-PEERs (PEERs average = 2.41, non-PEERs average = 2.79).

**Table 2:** Summary of the academic performance of PEERs and non-PEERs for Instructor *J* across 5 sections of the same course.

Section	PEERs	Mean (SD)	n	ΔGP	Test	
					t	p-value
1	No	2.70 (0.99)	222	-0.22	-1.93	0.0552
	Yes	2.48 (0.84)	89			
	Total	2.63 (0.95)	311			
2	No	2.63 (0.97)	157	-0.01	-0.11	0.9152
	Yes	2.62 (0.87)	66			
	Total	2.62 (0.94)	223			
3	No	2.79 (0.95)	272	-0.38	-3.93	0.0001
	Yes	2.41 (0.97)	147			
	Total	2.66 (0.97)	419			
4	No	2.88 (0.95)	261	-0.39	-3.83	0.0002
	Yes	2.49 (0.94)	129			
	Total	2.75 (0.96)	390			
5	No	2.95 (0.79)	133	-0.53	-4.68	<0.001
	Yes	2.42 (0.91)	104			
	Total	2.72 (0.88)	237			
Average				-0.31		

The mean and standard deviation of grades for Instructor *J* for non-PEERs and PEERs and the results of each of the independent samples *t*-test. The Grade Point Difference (ΔGP) between PEERs and non-PEERs is also displayed.

To determine how consistent these results were across multiple sections of the same course taught by a single instructor, we performed these same statistical analyses across five sections of the course (Tables 1 and 2). For this particular instructor, there was a statistically significant racial opportunity gap present during 3 of the 5 times the class was taught as determined both by comparing the distribution of A and B grades (Table 1), and the difference in GPA (Table 2) for PEERs and non-PEERs. The opportunity gap in GPA between PEERs and non-PEERs varied across the 5 sections, but was consistently negative (course-instructor average ΔGP = −0.31). The gap in the percentage of PEERs receiving A and B grades and the percentage of non-PEERs receiving A and B grades also varied across the 5 sections (course-instructor average Δ%AB = −13%; see Table 1).

**Fig 1:**
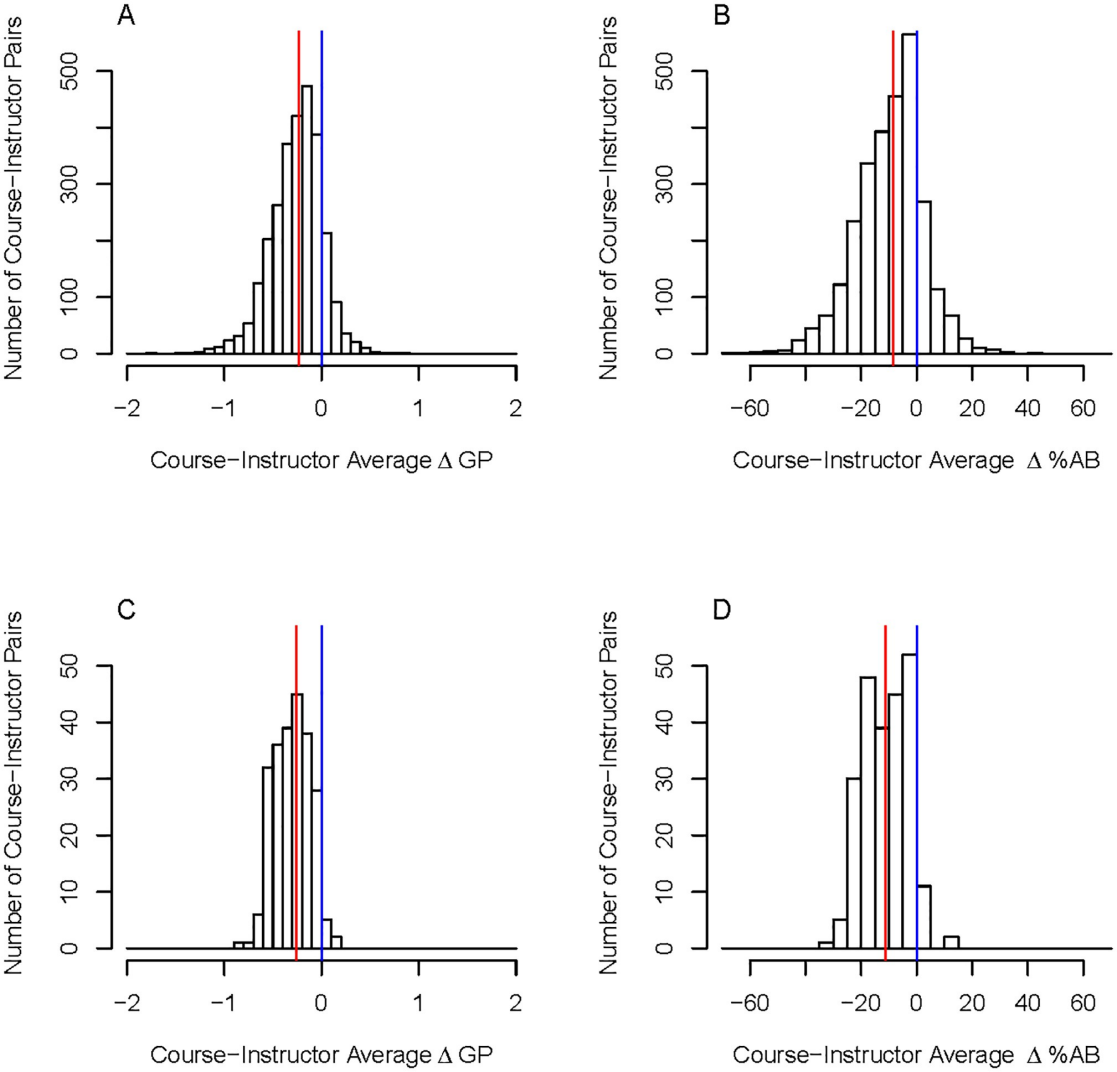
Histograms of the opportunity gaps between PEERs and non-PEERs per course section at the instructor level (course-instructor pairs). Histogram of the course-instructor pairs average grade point difference (ΔGP) and the average difference in the percent of A and B grades given to PEERs and non-PEERs (Δ%AB). The red line represents the median on each plot and the blue line represents no difference between PEERs and non-PEERs. (A) and (B) include all 2,752 course-instructor pairs. The median course-instructor average for ΔGP is -0.23 and for Δ%AB is -9%. (C) and (D) include course-instructor pairs that had at least 4 occurrences (233 such pairs). For these pairs with at least 4 occurrences, the median course-instructor average for ΔGP is -0.30 and for Δ%AB is -11%.

We now expand these analyses across all course-instructor pairs within our dataset. Fig 1 displays the course-instructor average ΔGP and course-instructor average Δ%AB between PEERs and non-PEERs. Fig 1A and 1B include all course-instructor pairs whereas in Fig 1C and 1D each instructor represented taught a minimum of 4 sections of a single course, enabling us to focus on those that are offered on a more regular basis. The median course-instructor average ΔGP across our sample was −0.23, which equates to nearly a quarter point GPA drop for PEERs relative to non-PEERs. When looking at the course-instructor average Δ%AB, there is a considerable spread among our sample (median value of −8.52%). Using this methodology, we were able to identify that 59% of the instructors had zero course sections with a significant difference between grades earned by PEERs and non-PEERs. However, if we only consider instructors who have taught multiple sections the percentage of instructors whose course sections never exhibited an racial opportunity gap decreases as the number of course sections taught increases; for instructors who taught 2 course sections the percentage is 54%, 3 course sections the percentage is 42%, and for instructors who taught at least 4 course sections the percentage drops to 19%.

### Research question 2: Are certain STEM departments more likely to exhibit racial opportunity gaps versus others?

**Fig 2:**
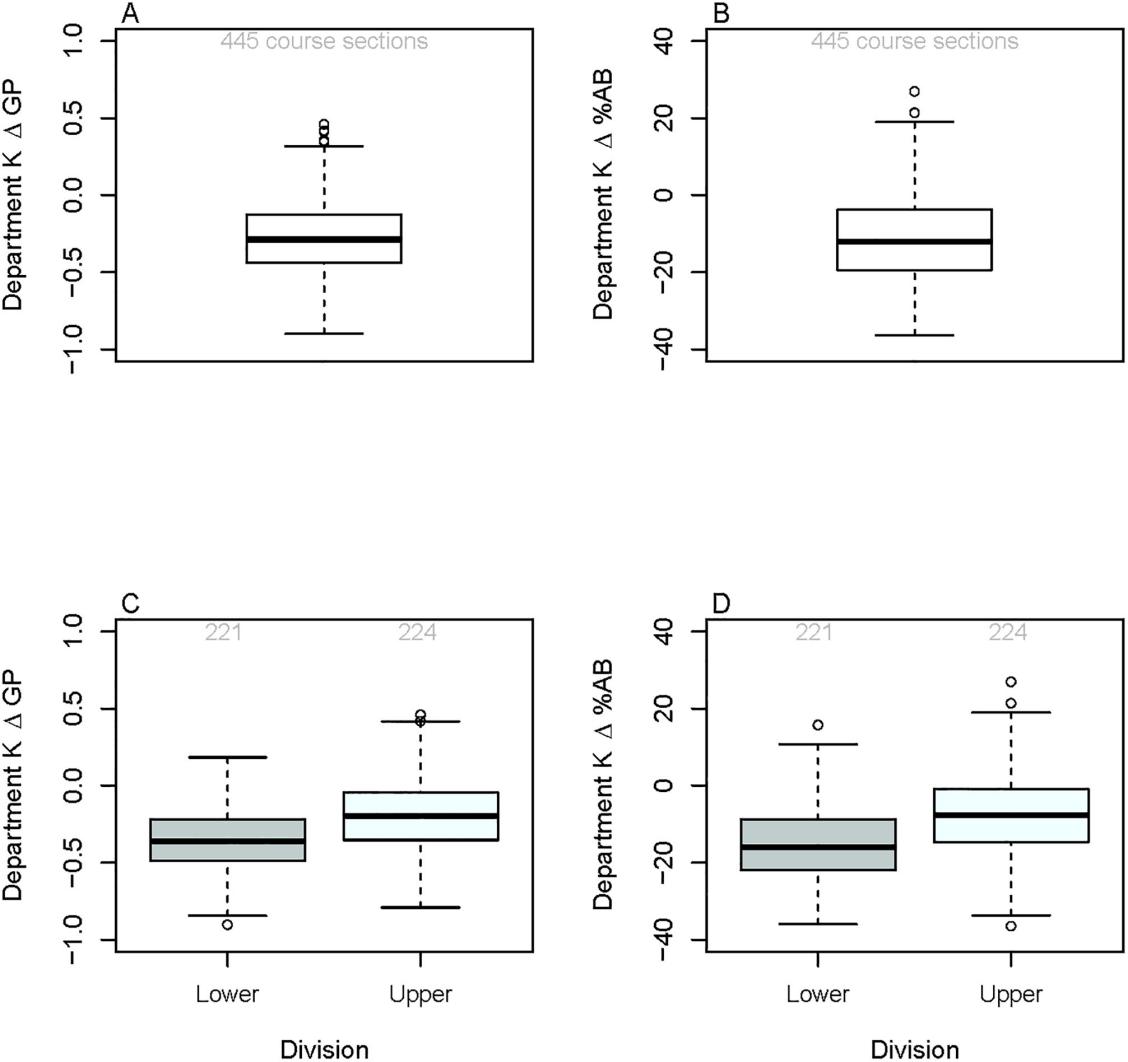
Boxplot of ΔGP and Δ%AB per course section for Department *K*. (A) and (B) combine the information from all course sections whereas (C) and (D) separate the course sections by level (lower versus upper division). A total of 445 course sections are displayed consisting of 74 unique courses, 189 unique instructors, and 217 unique course-instructor pairs. The 221 lower-division course sections had 24 unique courses, 96 unique instructors, and 107 unique course-instructor pairs. The 224 upper-division course sections had 50 unique courses, 102 unique instructors, and 110 unique course-instructor pairs.

Fig 2 provides an example of one departments’ (Department *K*) ΔGP and Δ%AB. Typically, PEERs received between -0.44 and -0.13 grade points lower than non-PEERs in course sections taught within Department *K*. The median Δ%AB in this particular department was -12%. For this particular department, there were 445 course sections included in the departmental analysis. The 445 course sections consisted of 74 unique courses, 189 unique instructors, and 217 unique course-instructor pairs. Of those course sections, 36% showed a difference in the distribution of A and B grades compared to C, D, and F grades for PEERs and non-PEERs. 47% showed a difference in the GPA received by PEERs and non-PEERs and 36% of the course sections in Department *K* showed a difference in both statistical tests.

**Fig 3:**
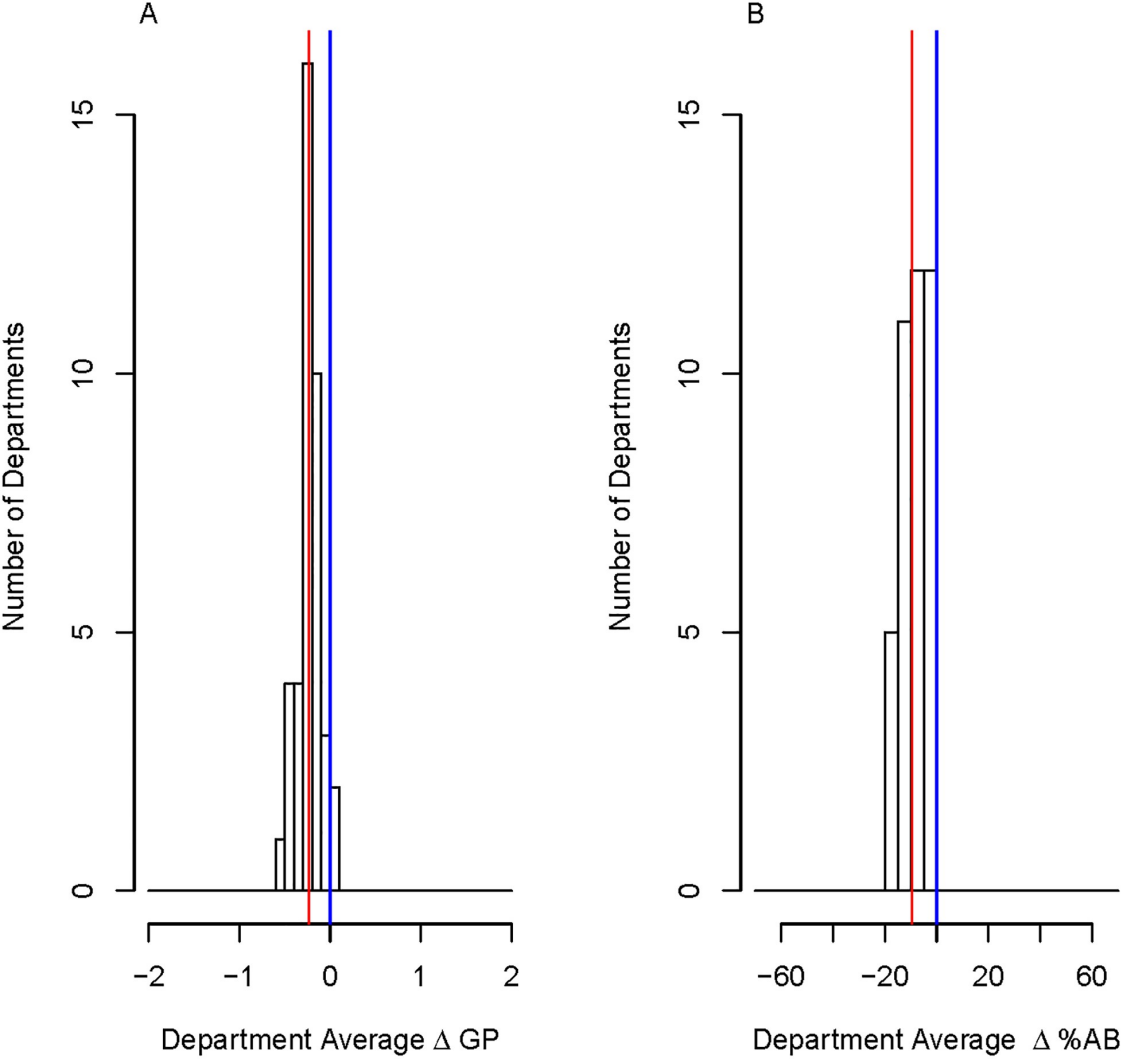
Histograms of the differences in grades (department average ΔGP and Δ%AB) given to PEERs and non-PEERs per course section at the department level. A total of 40 STEM departments are included in the plots. The red line represents the median on each plot and the blue line represents no difference between the two groups. The median STEM-department average for ΔGP is -0.24 and for Δ%AB is -9%.

We then conducted this same analysis for all STEM departments within the study institution to identify how consistent the racial opportunity gaps were across campus. Fig 3 displays the department average ΔGP and department average Δ%AB. The median STEM-department average ΔGP is −0.24 and the median STEM-department average Δ%AB is −9.45%, similar to our analysis looking at course-instructor pairs across campus (Fig 1). The median percentage of a given department’s course sections with statistically significant racial opportunity gaps was 15%. We were able to identify 1 exemplar STEM departments which had no course sections with a difference in grades given to PEERs and non-PEERs. This department had 9 course sections (that met our inclusion criteria) that were taught by 5 unique instructors and represented 5 unique courses. At the other extreme, there was one STEM department which we identified differences in grades given to PEERs and non-PEERs in 65% of their course sections. This department had more than 80 course sections consisting of 3 unique courses, 14 unique instructors, and 18 unique course-instructor pairs. This analysis would enable us to identify particular departments that may benefit from receiving targeted support to decrease racial opportunity gaps. Furthermore, in our particular dataset, we highlight exemplars; there were 55 instructors, 40 course-instructor pairs, and 69 courses who consistently did not have significant ΔGP in their course sections.

### Research question 3: Do racial opportunity gaps vary within a STEM department?

While it is clear that there is considerable variation in the size of racial opportunity gaps in course sections taught across departments within a single institution, we were curious to see how variable these gaps were within a single department. Fig 2 provides an example of the differences in grades received by PEERs compared to non-PEERs for each course section taught in Department *K* disaggregated by course level (lower versus upper division). The 221 lower-division course sections had 24 unique courses, 96 unique instructors, and 107 unique course-instructor pairs. The 224 upper-division course sections had 50 unique courses, 102 unique instructors, and 110 unique course-instructor pairs. There was significantly larger gaps for lower division courses compared to upper division courses for Department *K*’s average ΔGP (*t* = −7.80, *p*< 0.001) and average Δ%AB (*t* = −7.98, *p*< 0.001). While the overall *K*th department average ΔGP was −0.28, this value was −0.36 for lower-division course sections compared to −0.19 for upper-division course sections. The difference in the percentage of PEERs and non-PEERs receiving A and B grades were larger for lower-division courses compared to upper-division courses (lower-division-department average Δ%AB = −15%, upper-division-department average Δ%AB = −7%).

**Fig 4:**
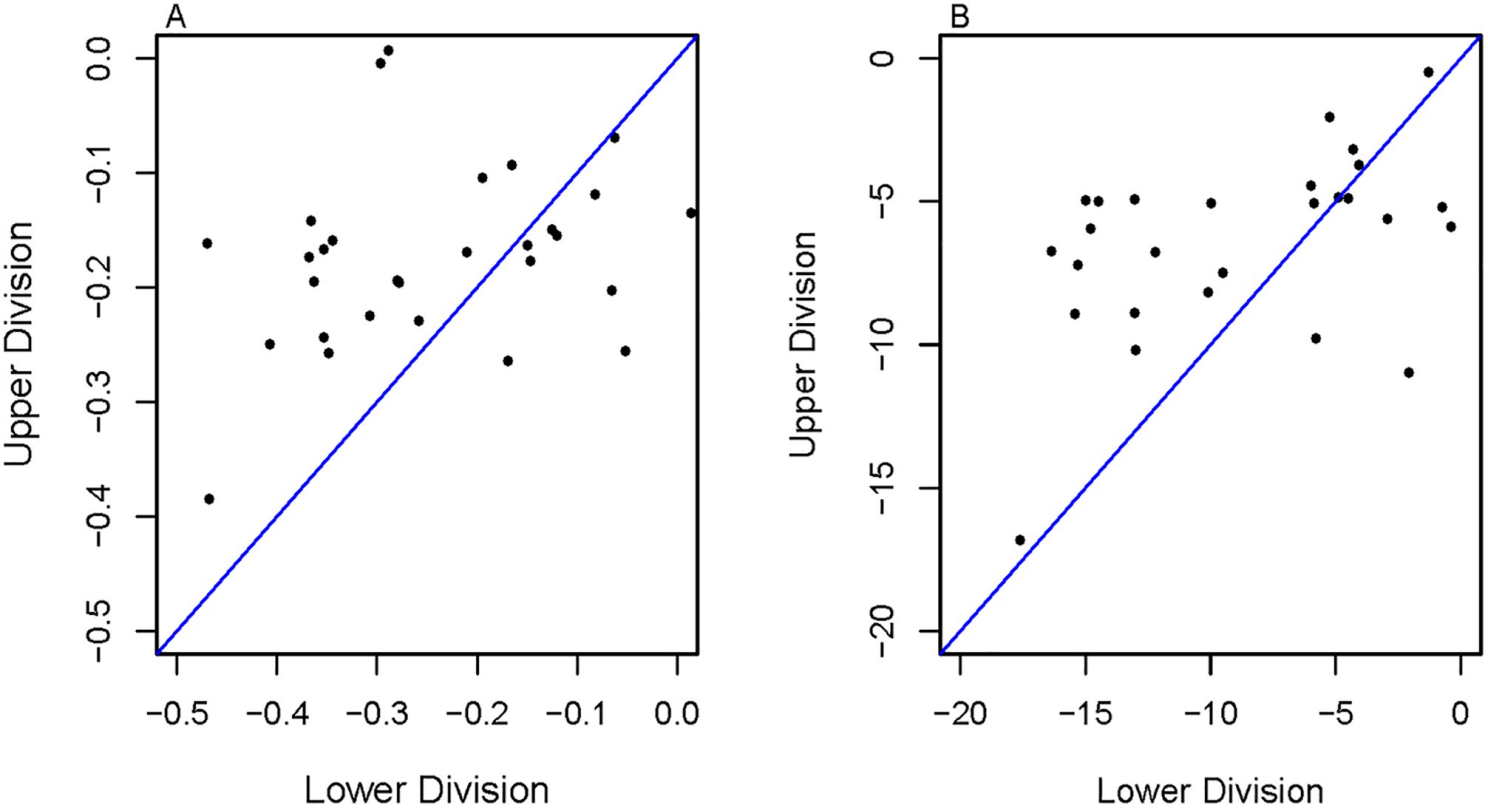
Scatterplots of the differences in grades given to PEERs and non-PEERs in lower and upper division courses at the department level. The plot on the left (A) is for department average ΔGP and on the right (B) is for department average Δ%AB. A total of 31 STEM departments had both lower-division and upper-division course sections and are included in the plots. The blue line (*y* = *x*) represents similarity in grades for lower and upper division courses (points above the line represent departments that have larger racial opportunity gaps in lower-division course sections).

Table 3 presents the opportunity gaps for different units (course sections, courses, instructors, course-instructor pairs, and departments). Out of the 40 (*n*_*K*_) STEM departments, we examined 20 (*n*_K*_) that taught a minimum of 10 lower-division and 10 upper-division course sections. The upper-division department average ΔGP (-0.19) is smaller compared to lower-division department average ΔGP (-0.25). The scatterplot in Fig 4 highlights that the lower-division course sections within a department generally had larger racial opportunity gaps (i.e. wider/larger department average ΔGP and Δ%AB, respectively) compared to upper-division course sections. There were only a handful of departments where the racial opportunity gaps were higher for the upper-division course sections compared to the lower-division courses.

**Table 3:** The number of lower-division (LD) and upper-division (UD) course sections, courses, course-instructor pairs, and departments as well as the respective summary statistics for racial opportunity gaps.

Unit of Analysis	Description	Number (%)	ΔGP	Δ%AB
Course Sections	LD	2698 (58%)	-0.33 (0.28)	-13 (12)
	UD	1946 (42%)	-0.19 (0.25)	-7 (12)
	Total	4644	-0.27 (0.28)	-10 (12)
Courses	LD	294 (32%)	-0.28 (0.23)	-10 (10)
	UD	611 (68%)	-0.18 (0.21)	-6 (10)
	Total	905	-0.21 (0.22)	-7 (10)
Course-Instructor Pairs	LD	1476 (54%)	-0.32 (0.27)	-12 (12)
	UD	1276 (46%)	-0.19 (0.23)	-6 (11)
	Total	2752	-0.26 (0.26)	-10 (12)
Course-Instructor Pairs (≥ 4 course sections)	LD	155 (67%)	-0.35 (0.19)	-13 (8)
	UD	78 (33%)	-0.22 (0.14)	-8 (6)
	Total	233	-0.30 (0.19)	-11 (8)
Instructors	LD	1124 (57%)	-0.30 (0.25)	-12 (12)
	UD	1012 (51%)	-0.19 (0.23)	-7 (11)
	Total	1983	-0.25 (0.25)	-9 (12)
Departments	LD	37 (93%)	-0.28 (0.15)	-10 (7)
	UD	34 (85%)	-0.19 (0.17)	-7 (6)
	Total	40	-0.24 (0.13)	-9 (5)
Departments (≥ 10 LD and 10 UD course sections)	LD	20 (100%)	-0.25 (0.11)	-9 (5)
	UD	20 (100%)	-0.19 (0.07)	-7 (3)
	Total	20	-0.23 (0.08)	-8 (4)

The mean and standard deviation (in parentheses) for the difference in performance measured as the average difference (ΔGP) and the difference in the specific rates of A and B grades (Δ%AB) for PEERs compared to non-PEERs are given in the last two columns.

## Discussion

In this study, we set out to shift the focus from macro level institutional data analysis to micro level data analysis at the course, instructor, and department levels, with the intention of gaining a better understanding of racial opportunity gaps and how they are distributed across the institution. The results from this particular institution show that PEERs are on average receiving lower grades compared to non-PEERs. Our results show that the racial inequities are larger for lower-division compared to upper-division course sections and these systemic inequities are consistent at many different levels (course-section, course, course-instructor pairs, instructor, and department). This consistent trend across the different levels of aggregation highlights the systemic nature of racial inequity.

The distribution of gaps across course sections, the existence of few instructors (who taught multiple sections of the same course) without gaps, and the low number of unique courses without gaps all point to the overlapping structural influences of instructor, course level, and department that result in gaps observed at the institutional level. The clustering of gaps around specific course sections or disciplines versus a uniform distribution across the curriculum is consistent with the evidence of larger systemic issues that research has been documenting for years in higher education. Due to factors like systemic bias [26, 27, 32], unequal power structures [2, 4, 25, 27, 31], conflicting cultural models [4, 27, 29, 32], and the internalization of expectations [25, 26, 28, 33], PEERs are denied access to the same type of success as non-PEERS.

There are limits, however, to what these data can reveal about racial opportunity gaps at an institution. To begin with, we did not incorporate student demographics besides ethnicity, such as gender or first generation status, or prior academic achievement when examining course section opportunity gaps. It is common practice to control for various student characteristics when conducting similar analyses [4, 7, 31], yet there are also many instances of utilizing raw score when institutions report these data [4, 33, 41]. We argue that not including student demographics and prior achievement in an analysis model more accurately captures the student experience, as undergraduates in a course do not consider their demographic characteristics or prior academic achievement when considering their course outcomes relative to their classmates. While we recognize that prior academic achievement often correlates with academic success in higher education [42–46], national statistics highlighting the commonly observed “achievement gap” similarly examine course grades, cumulative GPAs, and graduation rates in the absence of controlling for a measure of prior performance [47–49]. And future opportunities that reference a student’s academic performance, such as graduate school admissions or job interviews, also consider only the raw performance. Another limitation is that our analyses do not extend beyond student institutional data, and thus do not consider course structures, classroom practices, or instructor level characteristics. These are likely critical factors that impact the presence of a racial opportunity gap, so it is important the future work enables us to add these data to our models. And from an analytical perspective, since the distribution of grades at the course section level tends to be skewed, we used two different tests to triangulate our results. This produces a conservative estimate of differences in opportunity between PEERs and non-PEERs. Thus, we likely have identified only the lower bound of racial opportunity gaps present across an institution.

By examining these data on an institutional level, we were able to identify interesting and perhaps unexpected patterns. One is the finding that the presence or absence of racial opportunity gaps within a given course for a given instructor were not consistent. The majority of instructors in our dataset had some course sections where a racial opportunity gap was present while other instructors where it was not. This perhaps highlights the variability in instructor practices. In support of this, Owens et al. [50] demonstrated using classroom audio analyzed by the Decibel Analysis for Research in Teaching (DART) algorithm that a course section varies considerably from lecture to lecture in regard to the amount of lecture implemented. It is not a stretch to assume that examining a given instructor’s lecture periods across distinct offerings of a course would result in variability as well. While core structural and pedagogical approaches may remain the same for faculty, their presentation, degree of buy-in sought from students, and communication around course policies can still vary among iterations of a given course highlighting the importance of examining multiple iterations of a given course offering, as we did in this analysis.

Another reason for variability between the course-instructor pairs may be due to varying demographics of the course staff and students [51–53]. For example, having a PEER teaching assistant may increase belonging and decrease imposter syndrome. From the student population perspective, while student demographic characteristics in our dataset were fairly consistent from section to section, differences may exist that do not manifest themselves as easily as ethnicity or gender, including non-cognitive factors like sense of belonging or growth mindset [4–6, 25–27, 29, 32]. And while we focused on the percentage of PEERs in a course section, the total number of PEERs in a course may create a higher sense of belonging (for example 2 PEERs in a class of 10 versus 20 in a class of 100), so course enrollment may play a role as well [25, 26, 41].

We also found that the prevalence of racial opportunity gaps within an institution is not consistent across STEM programs. This is perhaps not surprising considering that student demographics vary across STEM programs. For example, Yang and Barth looked at the distribution of male and female students in STEM majors at two different public universities, one in the Southeast and the other in the Midwest. An analysis of 1,848 students showed that biology tends to have a larger representation of women when compared to other STEM majors like computer science, engineering, mathematics, and the physical sciences including physics, geology, and chemistry [54]. In another study, Riegle-Crumb and King utilized data from the Educational Longitudinal Study (ELS) of 2002 to follow a cohort of approximately 15,000 students who were enrolled in a 4-year degree-granting institution in 2006. They found that Black males make up 28.5% of physical science/engineering majors while Black females make up only 7.3% of physical science/engineering majors. Comparatively, biology is much more gender balanced, with Black males making up 7.0% of majors and Black females making up 8.6%. This trend holds true for white and Latinx males and females [55].

And our data, perhaps not surprisingly, revealed that lower division courses were more likely to exhibit racial opportunity gaps relative to upper division courses within a given STEM department. This is supported by considerable literature highlighting the gatekeeper nature of lower division courses [4, 25–27, 32] and may also reflect that the disproportionate loss of PEERs in these courses leads to a more homogeneous upper division undergraduate population in terms of prior academic achievement. But it may also be a factor of differing grading practices in courses of these two levels. The lower division courses are more likely to use norm-referenced grading, where regardless of overall achievement, a fraction of the course section is destined to earn a C or lower [56–58]. From the instructor and institution perspective, we would argue that there is not a pedagogically-beneficial reason to utilize distinct course policies for lower and upper division courses, and plan to examine whether these are contributing to these differing racial opportunity gap outcomes.

In the rapidly changing higher education landscape, the increased use of data in higher education to evaluate successes and failures enables administrators to make decisions in an empirical fashion. Our analysis can be replicated within one’s own institutional context to show the persistence of racial opportunity gaps, but it is important to note that it does not provide an explanation as to what is causing these gaps. In our particular dataset, we highlighted exemplars. By providing a way to identify these exemplar instructors, course-instructor pairs, courses, and departments, a more detailed, qualitative examination can help to better identify the practices and policies that result in these more equitable outcomes.

## Supporting information

S1 Data (XLSX)
